# Durvalumab as Consolidation Therapy in Post-Multimodal Interventional Treatment for Patients with Advanced Solid Tumors: A Preliminary Study

**DOI:** 10.1155/2022/7794840

**Published:** 2022-03-15

**Authors:** Yuanming Li, Runqi Guo

**Affiliations:** Minimally Invasive Tumor Therapies Center, Beijing Hospital, National Center of Gerontology, Institute of Geriatric Medicine, Chinese Academy of Medical Sciences, Beijing, China

## Abstract

With 2.1 million unique cases of lung tumors and 1.8 million mortalities in China, advanced solid tumors continue to be the primary source of cancer mortality rates. Nearly two-thirds of lung cancer individuals display advanced-stage tumors at the time of testing, with a 5-year survival ratio of 7%. People with advanced solid tumors have an appalling outcome, with a 5-year total survival ratio of roughly 15%. Immunotherapy inhibitors, like those for programmed cell death protein 1 (PD-1) and programmed death-ligand 1 (PD-L1), have ushered in a novel period in cancer diagnosis and therapy. Three resistant medications were authorized for advanced solid tumors: nivolumab, pembrolizumab, and atezolizumab. Durvalumab, an anti-PD-L1 antigen, is currently being researched. Durvalumab's pharmacologic characteristics, clinical efficacy, and security as consolidation therapy in post-multimodal interventional therapies for people with advanced solid tumors are discussed in this paper. We have also shared details of two patients who were identified with advanced solid tumors and were provided with durvalumab medication. The performance measures like Progression-Free Survival (PFS), Overall Survival (OS), and Overall Response Rate (ORR) are also contrasted for different antibodies. The research findings imply that durvalumab consolidation therapy is a cost-efficient therapy, while health policymakers should address the financial consequences.

## 1. Introduction

Lung cancer death proportions in both males and females in China have outpaced all other tumors for nearly three decades. Such higher mortality rates are mostly due to a mixture of higher lung tumor occurrence and weak durability results for people with lung tumors discovered in later phases. Patients with distant metastases have a five-year survival rate of about 5%. Even with therapies, sophisticated lung cancers are rarely curable; instead, the aim is to slow the spread of the disease and promote healthy behaviors. Recent improvements in immunotherapy have resulted in significant advancements in the therapy of solid cancer tumors. In the first-line and second-line situations, monoagent PD-1/PD-L1regulators and immunotherapy with chemotherapeutic mix treatments have shown advancements in overall survival (OS). Following the introduction of such medications as conventional regimens in the first and second lines, attempts have been made to shift their employment sooner in the course of disease [1].

In randomized stage II and III medical studies, immune checkpoint inhibitors (ICIs), a unique category of medications, are exhibiting promising outcomes in people with severe non-small-cell lung cancer (NSCLC). Anti- PD-1 and anti- PD-L1 are examples of ICIs. The anti-PD-1 antigen nivolumab was the initial ICI to be shown to be a successful second-stage therapy in two-stage III studies in NSCLC people. Pembrolizumab, an alternate anti-PD-1 antigen, is a successful first-stage treatment for people whose cancers have a PD-L1 positive of 50% or more. Individuals who obtained pembrolizumab had an average PFS of 10.4 months, contrasted to just 6.0 months in those who received chemotherapies. The anti-PD-L1 antigen atezolizumab was found to be effective as a second-stage therapy, with an average overall survival rate of 13.8 months contrasted to 9.6 months with docetaxel. Such findings may help to solve the dismal prognosis of unresectable phase III NSCLC, regardless of the prevalence of traditional multimodal therapy, by introducing innovative diagnostic approaches, which include immunotherapy [2].

The most researched ICIs include CTLA4, PD-1, and PD-L1 inhibitors that have displayed effectiveness in enhanced stage solid tumors such as NSCLC. Durvalumab (previously termed MEDI4736) is a humanized monoclonal IgG1*κ* antigen that focuses on PD-L1. Durvalumab attaches to the PD-L1 receptors found on tumor tissues with higher affinity and specificity. Durvalumab recovers T-cell cytotoxic activity by blocking the association between PD-L1 and its ligands presented on immune tissues. In addition to preventing the PD-L1/PD-1 association, PD-L1 is represented on effector T tissues, and reports have highlighted that PD-L1-inhibiting antigens can activate the PD-L1 intracellular signaling pathway in CD8+ T tissues. This intracellular signaling pathway is prone to causing T-cell death, resulting in a paradoxical consequence [3].

For progressive NSCLC, addressing ICIs is a conventional therapeutic approach. Immunotherapy methods, like inhibitory components essential for breaching an antitumor immune reaction, like PD-1 and programmed cell death ligand 1 (PCDL1), are developed as a result of a finer knowledge of the immunological nature of NSCLC. Nivolumab, pembrolizumab, and atezolizumab were authorized as therapeutic options for individuals who were already treated with enhanced solid stage tumors. Pembrolizumab is further approved as an initial-stage therapy for therapy-naive metastatic tumor people with PD-L1 transcription of more than 50% and metastatic tumor people with PD-L1 transcription of more than 1% after advancement following first-stage platinum-dependent doublet therapy. Furthermore, the authority has recently approved pembrolizumab in conjunction with platinum-dependent chemotherapies in unresectable enhanced NSCLC regardless of PD-L1 level, making it the benchmark of therapy in this context. Such findings could help to improve the dismal prognosis of phase III NSCLC patients by introducing innovative therapeutic approaches that include immunotherapy [4].

Durvalumab is a monoclonal antibody that is used to treat cancers of the lungs, bladder, and urinary system. Durvalumab is a cancer-fighting antibody that is now being tested in clinical studies for a range of disorders, including mesothelioma. When cancer has spread to other regions of the body and cannot be removed with surgery, or when conventional therapies have failed or discontinued functioning, durvalumab may be used.

Durvalumab, a unique ICI that functions as an anti-PD-L1 antigen, avoids PD-L1 from attaching to PD-1 and B7-1, permitting T tissues to detect and destroy cancer tissues. Durvalumab as consolidation treatment was found to have a better PFS than placebo in phase III NSCLC people who had not progressed following two or more rounds of platinum-dependent chemoradiation therapy (CRT). Durvalumab was authenticated by the FDA as consolidating chemotherapy in phase III illness after chemoradiation therapy, the first such case in the utilization of ICIs, based on these findings. Durvalumab was authorized by the EMA in September 2018 as a consolidating treatment in phase III illness following CRT, but only if PD-L1 is represented in greater than 1% of cancer cells [5].

This paper highlights the pharmacologic characteristics, clinical efficacy, and security of durvalumab as consolidation therapy in post-multimodal interventional therapies for people in China with enhanced solid cancers. The further part of the paper is arranged as follows: Section 2 describes the properties and characteristics of the antibody ‘durvalumab,' and Section 3 analyzes the case study of two patients diagnosed with lung cancer who were treated with durvalumab. Also, the performance analysis of durvalumab compared with other therapeutic antibodies is provided in this section. Finally, part IV concludes the idea behind the paper.

## 2. Analysis of Durvalumab

### 2.1. Process of Action of Durvalumab

The PD-1 receptors, which are represented on stimulated T tissues following antibody exposures and have two ligands, PD-L1 and PD-L2, are immunological checkpoint proteins. PD-L1 is a transmembrane protein found in blood tissues, cancer tissues, and tumor stroma. CD80 (also known as B7-1, displayed on stimulated T tissues and antibody-presenting tissues) and PD-L1 can attach to each other. The relationships between PD-L1 and PD-1 may cause cytotoxic T tissue stimulation to be inhibited [6, 7].

PD-L2 governs the priming and polarization of T lymphocytes and is less commonly expressed in malignancy [8]. To present, there is no indication that antigens against PD-1that inhibit both PD-L1 and PD-L2 binding are more efficient than antigens against PD-L1 alone.

Durvalumab is a humanized immunoglobulin G1-kappa monoclonal antigen, which attaches to PD-1 and CD-80 and inhibits PD-L1. Durvalumab attaches to PD-L1 with a higher affinity but not to PD-L2, which controls inflammation in ordinary tissues; this method of activity may reduce the immune-associated damage correlated with the PD-L2 connection.

### 2.2. Clinical Efficiency of Durvalumab

#### 2.2.1. Durvalumab Is a Metastatic Disease

Durvalumab was initially tested on individuals with enhanced solid tumors, particularly refractory enhanced NSCLC individuals, in a large stage I/II research. Durvalumab was given intravenously (IV) at doses ranging from 0.1 to 10 mg/kg every two weeks or 15 mg/kg every three weeks (Q3W) for up to 12 months or till intolerable morbidity or advancement of the diseases occurred. Durvalumab 10 mg/kg was provided to 304 patients, with a total reaction rate of 17.5 percent. Treatment-naive patients had higher response rates (total reaction rate of 27.1 percent vs. 13.0 percent in extensively pretreated people) as well as while PD-L1 transcript was substantial (⩾25 percent PD-L), exhibiting a total reaction rate of 25.3 percent vs. 6.1 percent in PD − L1 < 25 percent PD-L.

#### 2.2.2. Durvalumab in Advanced Solid Tumors

When radiation is coupled with anti-PD-1, preclinical results consistently demonstrate a clear positive and potentially synergistic effect [9]. During the cancer progression, the relation between the tumors and the host resistant systems develops from immunological eradication, wherein the immune system recognizes and destroys cancer cells, to immunological stability, wherein cancerous tissues and the immune system cooperate, and ultimately immunological evasion. Upregulated suppressive ligands and cytokines, as well as diminished major histocompatibility complex (MHC) group I transcription, characterize the immune-escape phase that results in weak antibody representation and hides cancer from immune monitoring and eradication [10]. Irradiation may ‘uncover' cancer, allowing it more transparent to the inherent and flexible resistant responses, by activating downstream immune reactions and activating T cells, as well as upregulating MHC-I transcription on the cancer surfaces, allowing for better visualization of tumor-specific proteins (which improves the visibility of cancer to cytotoxic T tissues) [11].

### 2.3. Durvalumab Consolidation Therapy

The mainstay of treatments for advanced or metastatic stage III NSCLC is concurrent chemoradiation therapy (CRT). Durvalumab therapy after CRT is a significant therapeutic innovation that improves survivability in this sample of patients.

#### 2.3.1. Durvalumab Plus Tremelimumab

Despite the therapeutic improvement as seen in individuals administered with medications, which inhibit the PD-1/PD-L1 pathways, only around a fifth of individuals can have long-lasting reactions. As a result, efforts to boost T-cell activation are required to enhance results. Preclinical evidence suggests that CTLA-4 and PD-(L)1 medications in conjunction possess a synergic activity through separate modes of action. To put it another way, inhibiting CTLA-4 allows CD28 to attach to CD80/CD86, enhancing the coactivation signals needed for rigorous T tissue stimulation and effector operation, whereas inhibiting PD-1 increases T tissue stimulation and cytotoxicity action on active and tired cells [12]. Even though such mixtures seem to be more hazardous, the most adverse reactions are resistance-associated serious impacts that shall be acute but are generally treatable with immunosuppressive drugs.

In melanoma and other tumor forms, namely NSCLC, inhibiting many immunological pathways instead of one has been demonstrated to have a better therapeutic effect in people [13, 14].

Tremelimumab is a CTLA-4 blocker, which stimulates the stimulation of cytotoxic T tissues in the early stages of the immunoreaction. It is a fully humanized IgG2 monoclonal antibody. Addressing the PD-L1/PD-1 and CTLA-4 pathways may exhibit synergistic or additive impacts, according to premedical research [15].

The first randomized accessible, multicenter, stage Ib medical trial of durvalumab and tremelimumab found that durvalumab 20 mg/kg and tremelimumab 1 mg/kg every 4 weeks seemed to have a controllable tolerability factor and anticancerous behavior irrespective of PD-L1 condition, and this concentration was chosen for the growth stage [16]. The most frequent adverse impacts were compatible with durvalumab and tremelimumab's recognized safety characteristics and were equivalent to the nivolumab and ipilimumab combos. The most prevalent drug-associated side effects were dysentery (11; 11 percent), colitis (9; 9 percent), and elevated lipase (9; 9 percent) in 42 percent of the entire cohort and 17 percent of the group having durvalumab 20 mg/kg and tremelimumab 1 mg/kg every four weeks (8; 8 percent).

#### 2.3.2. Durvalumab with Chemotherapy and Various Drugs

Chemotherapy's double involvement in cytotoxic and immunological stimulation has offered a scientific foundation for the creation of therapeutic mixtures. Just after the positive outcomes of medical studies, incorporating an anti-PD-1 with chemotherapeutics in enhanced solid tumors in the initial-stage configuration [17, 18] and an anti-PD-L1 with chemotherapeutics and bevacizumab in advanced solid tumors in the second-stage configuration [19], numerous studies are now looking into incorporating an ICI with chemotherapeutics to improve efficacy. The Canadian Cancer Trials Organization is evaluating the effectiveness and safety of the platinum-dependent chemotherapeutic treatment and durvalumab with or without tremelimumab in malignant tumors in a PD-L1 unselected cohort in a phase Ib trial. Durvalumab 15 mg/kg every three weeks and tremelimumab one mg/kg (multidosage q6w) or 3 mg/kg (3 dosages q6w) could be securely administered with complete dosages of platinum-doublet chemotherapeutic, according to the researchers. An ORR of 52.9 percent (95 percent CI: 28–77 percent) was observed in 17 of the 24 people with severe NSCLC. The maximum drug-associated adverse impacts were grade 1 or 2, allowing the dosage for the quadruplet treatment's scheduled stage II and III investigations to be determined [20].

Several trials were conducted to assess the feasibility of combining a TKI with ICIs, with increased response percentages recorded but greater morbidity. Two medical studies have looked at the toxicity profile of using a TKI in conjunction with durvalumab. The initial one is a stage I analysis combining gefitinib 250 mg once daily with durvalumab 10 mg/kg every two weeks, which revealed an overall response rate of 80% in twenty treatment-naive EGFR-mut tumor individuals. Diarrhea (80%), elevated ALT/AST (55%), and rashes (60%) were the most general treatment-associated negative impacts, with 4 patients discontinuing treatment. TATTON is a multiple arm stage Ib studies evaluating osimertinib in conjunction with various new medicines in people with severe tumor cells with EGFR mutation [21]. Eleven treatment-naive EGFR-mut tumor individuals were added in the growth stage following a dosage intensification stage of osimertinib 80 mg once daily with durvalumab 10 mg/kg every 2 weeks, with an overall response rate of 80% [22]. Despite this, interstitial lung illness was observed in seven among the eleven individuals (64 percent; comprising 3 grade 3/4 instances) and diarrhea in six of the 11 individuals (55 percent), leading to the study's premature termination due to pulmonary toxic effects [23].

Due to incidences of significant liver toxicity, a stage I/II research assessing the security and feasibility of nivolumab and crizotinib for the initial-stage therapy of ALK-positive enhanced tumors had to be stopped early. In ALK-positive advanced NSCLC individuals, a stage I/II medical study is assessing the effectiveness of a novel ALK TKI (dasatinib) in combination with durvalumab [24].

#### 2.3.3. Durvalumab with Radiotherapy

The PACIFIC clinical trial's outstanding outcomes have laid the groundwork for many medical studies integrating irradiation and durvalumab, including the PACIFIC-2 research, which will investigate a concurrent chemoradiation and the durvalumab group. In individuals with phase IIIa NSCLC, the effectiveness of durvalumab with or without tremelimumab is given every four weeks for 2 dosages along with conventional thoracic radiotherapy (RT) as preoperative immunoradiation has now been studied. A study evaluating the efficiency and feasibility of durvalumab with or without SBRT in individuals with phase I–IIIa NSCLC is also ongoing in the preoperative context. Many current medical studies for individuals with oligometastatic and metastatic diseases are assessing the therapeutic efficacy and security of durvalumab with or without tremelimumab paired with various dosages and regimens of radiotherapies depending on the abscopal impact concept [25].

#### 2.3.4. Biomarkers

Biomarker transcription is a significant problem that must be considered whenever contemplating tailored treatments to reduce extraexposures to possible toxicity while also ensuring maximum efficacy. The transcription of PD-L1 on tumor tissues is a prospective marker for the efficiency of PD-1/PD-L1 immunotherapies, and it is frequently utilized to guide the clinical decision. PD-L1 transcription, on the other hand, did not completely anticipate the responsiveness to PD-1/PD-L1 ICIs. Instead, in numerous tumor forms, particularly NSCLC, a higher mutational load has been linked to a better response to immunotherapeutics. Furthermore, the latest studies of the CheckMate 568 and CheckMate 227 studies found that the use of the TMB to determine whether customers are more likely to gain from nivolumab with ipilimumab is critical. Likewise, bTMB was found to be a possible marker for choosing individuals for durvalumab plus tremelimumab treatment in a premeditated exploratory study of the MYSTIC research. Durvalumab with tremelimumab produced a longer-lasting reaction and a greater 2-year OS percentage in patients who are at high TMB than those with a lower TMB in that research. Nevertheless, the unfavorable findings of the current stage III research (NEPTUNE), wherein the primary outcome was not fulfilled in individuals with blood TMB 20 ≥ mut/Mb, were unsatisfactory [26].

One of the most investigated indicators for ICIs is the transcription of PD-L1 in malignant cells. In several tumor types, this transcription is associated with a poor prognosis, but in several medical tests, particularly NSCLC, a straight proportionate link among PD-L1 transcription and the effectiveness of PD-1/PD-L1 pathway blockers has been demonstrated. Nonetheless, when chemotherapy was added to immunotherapeutic or 2 distinct ICIs were combined, the advantage ineffectiveness was replicated in every subset of patients, irrespective of PD-L1 transcription in cancerous cells, though the significance of the advantage appears to be still mainly connected to PD-L1 transcription. Several IHC tests for PD-L1 transcription, on the contrary, were authorized in tandem with various treatments, every one designed by a different organization and utilizing a different cut-off for the transcription of these biomarkers. Numerous unanswered questions complicate the use of PD-L1 IHC as a predictive biomarker: changeable identification receptors, different IHC cut-offs, cell preparation, processing variance, main vs. metastasis biopsy specimens, oncogenic vs. stimulated PD-L1 transcription, and staining of tumor cells vs. immune cells. This makes it impossible to exchange the existing tests and cut-offs in every site for the various drugs available, which may result in certain individuals' PD-L1 status being misclassified.

### 2.4. Clinical Potential and Future Directions of Durvalumab

Individuals with chronic NSCLC now have more therapeutic approaches because of the immunological period, which has improved lifespan results while possibly reducing toxicities. Integrating an anti-PD-(L)1 with chemotherapeutics, one of the most recent techniques in NSCLC medical studies, has revealed persistent reactions and substantial changes in PFS and OS throughout all classes of PD-L1 transcription in cancer tissues. Provided the recent statistical link among TMB and immunological responses, as well as the fact that individuals with mutated EGFR and PD-L1 could benefit from durvalumab and other immunotherapeutic treatments, the quest for an accurate forecasting biomarker is among the most pressing concerns that must be addressed. On the other hand, understanding the biology of nonresponder cancers and also the causes for progression of the disease in first respondents is critical. More specifically, durvalumab has demonstrated long-term therapeutic value in progressed NSCLC patients who have PD-L1 transcription in less than 25% of cancer cells. Durvalumab's PFS was considerably longer in stage III NSCLC individuals following chemoradiotherapy (16.8 months) compared to placebo (5.6 months). In addition, the average time to metastatic disease or mortality was similarly enhanced in the experimental part of the research. Although the anticipated risk of cancer was enhanced, it was only of a smaller extent and was easily controlled in most cases. Several medical studies with durvalumab as monotherapy and in conjunction with other medicines are now underway, with tremelimumab being among the most potential collaborators for durvalumab. To contrast its efficiency to the standard of care chemotherapy, findings from two stage III trials in the first-stage scenarios are needed. Other areas of research have included the prospect of treating patients with chemotherapy and radiotherapy that might possibly modify PD-1 and PD-L1 expression levels in cancers. ICIs like durvalumab and others that are now being studied in NSCLC individuals may assist in determining their potential advantages.

## 3. Case Study Analysis

12 patients from Minimally Invasive Tumor Therapies Center, Beijing Hospital were arbitrarily assigned to obtain durvalumab consolidation treatment after being identified with advanced solid tumors. The trial was approved by the Ethics Committee of Minimally Invasive Tumor Therapies Center, Beijing Hospital, and patients or their families gave informed consent. Written informed consents were obtained from the patients or families before inclusion. Durvalumab (at a dosage of 10 mg per kg of body weight intravenously) was given to individuals in a 2 : 1 ratio every two weeks for up to 12 months. The trial medicine was given to the participants 1 to 42 days after they had chemoradiotherapy.  Table 1 shows the characteristics of patient data.

Molecular markers are DNA fragments that serve as genetic indicators for detecting changes in gene sequences, protein expression levels, and protein structures and activities. Genomics allows for a more complete study of cancer, it aids in tumor molecular identification. The normal range of molecular data is 2.5 ng/ml. Based on the type of experiment used, the normal range may differ somewhat. Levels more than 10 ng/ml indicate widespread illness, whereas levels greater than 20 ng/ml indicate metastatic disease. PFS, OS, and ORR are the performance measures. Two case reports are explained in detail below.

**Figure 1:**
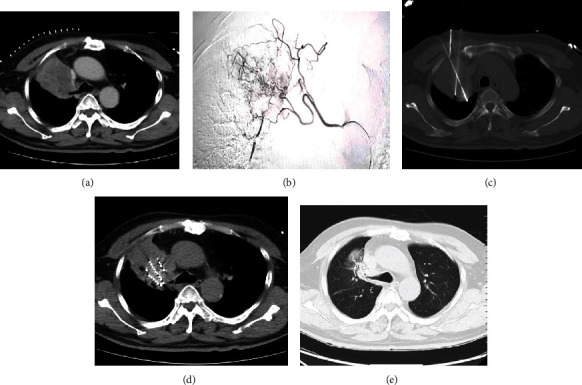
(a) An enhanced CT scan of the chest, a lump in the upper lobes of the right lung, with uneven enhancement. (b) Bronchial arteriography, showing obvious abnormal tumor staining. (c) The microwave ablation needle and the needle cloth needle for seed implantation. (d) The scanned image immediately after microwave ablation and seed implantation. (e) The reexamination of chest CT scan in 2021 December. The lesions were significantly reduced and the particles were gathered satisfactorily.

A 64-year-old man with right lung squamous cell carcinoma cT4N3M1a stage Iva, with no surgical opportunity, refused systemic chemotherapy and came to the hospital due to hemoptysis in 2020-11. Enhanced CT revealed a lump in the upper lobes of the right lung (maximum cross-section about 7.8 × 7.5 cm), showing uneven enhancement. The lesion and the pleura were unclear, and the upper lobe of the right lung bronchus was truncated with atelectasis. Two courses of bronchial artery drug-loaded microspheres chemoembolization (recombinant human endostatin 90 mg, nedaplatin 80 mg infusion chemotherapy, and gemcitabine 800 mg loaded with 300-500 *μ*m CalliSpheres drug-loaded microspheres for embolization). In 2021-02, the right lung tumor was treated with microwave ablation and simultaneous iodine 125 radioactive seed implantation (50 pieces of 0.6 mCi). Follow-up regular immunotherapy with varizumab 1000 mg Q4W was provided. Review and evaluation were carried out PR in 2021-11 (Figure 1).

**Figure 2:**
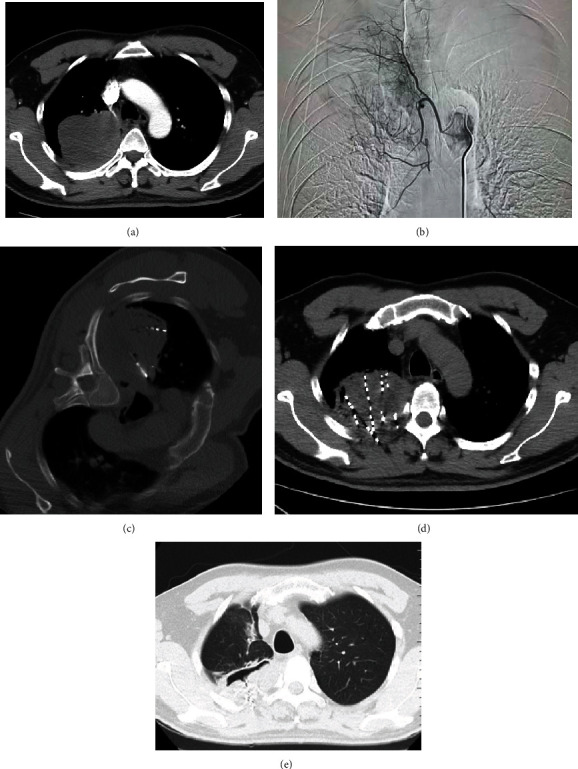
(a) An enhanced CT scan of the chest, with a lump in the upper lobes of the right lung, with enhanced edges. (b) Bronchial arteriography, showing obvious abnormal tumor staining. (c) The microwave ablation needle cloth needle and seed implantation. (d) The scanned image immediately after microwave ablation and seed implantation. (e) The chest CT plain scan for the evaluation in 2021 September. The lesions were significantly reduced, necrotic cavities were formed, and the particles were gathered satisfactorily.

A 49-year-old male with poorly differentiated right lung cancer cT4N3M0 stage IIIC refused surgery, radiotherapy, and chemotherapy, accompanied by obvious hemoptysis, came to our hospital in 2020-10. The enhanced CT scan of the chest displayed a mass in the upper lobes of the right lung (maximum cross-section 9.4 × 10.8 cm), with the main edge enhancement. Three courses of bronchial artery chemoembolization (recombinant human endostatin 90 mg, nedaplatin 80 mg, gemcitabine 400 mg infusion chemotherapy, and gemcitabine 800 mg loaded with 300-500 *μ*m CalliSpheres drug-loaded microspheres for embolization). In 2021-02, the right lung tumor was treated with microwave ablation and simultaneous iodine 125 radioactive seed implantation (60 pieces of 0.6 mCi). During the treatment period, regular vedolizumab 1000 mg Q4W immunotherapy was provided. Review and evaluation were carried out PR (per rectum) in 2021-07 (Figure 2).

**Figure 3:**
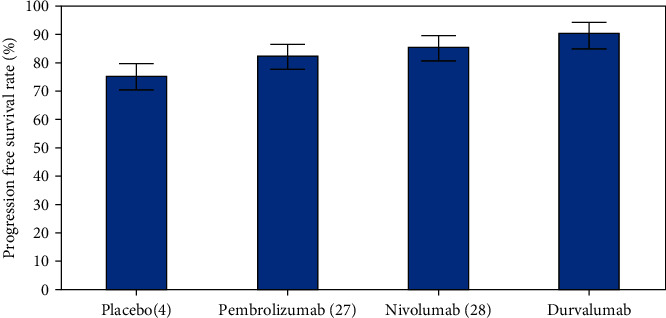
Comparative analysis of Progression-Free Survival (PFS) rate (%) for different antibodies.

**Figure 4:**
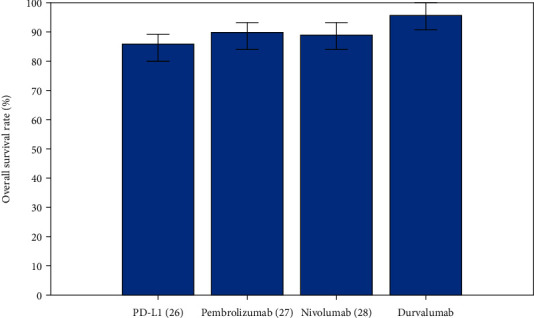
Comparative analysis of Overall Survival (OS) rate (%) for different antibodies.

**Figure 5:**
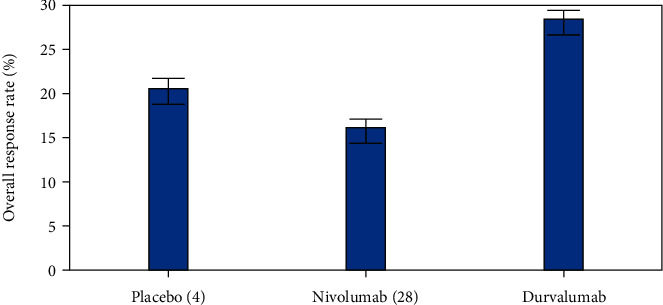
Comparative analysis of Overall Response Rate (ORR) (%) for different antibodies.

We have also compared the progression-free survival (PFS) rate, overall survival (OS) rate, and overall response rate (ORR) of different antibodies for individuals with advanced solid cancers as shown in Figures 3, 4, and 5, respectively. Durvalumab revealed a better performance when compared to other antibodies.

Progression-free survival (PFS) is the proportion of patients who did not develop new tumors or had cancer spread during or after therapy. The illness may have reacted totally or partially to therapy, or it may have remained stable. The cancer is still present, but it is not developing or expanding. In Figure 3, we have compared progression-free survival rate (%) for different antibodies namely Placebo (4), Pembrolizumab (27), Nivolumab (28), and Durvalumab. In the above figure, you can see that the PFS (%) rate of antibody durvalumab is higher than other antibodies.

Another word that is frequently used in conjunction with cancer treatment is overall survival. This represents the duration of time between the moment of diagnosis (or the commencement of therapy) till death. It is frequently used to determine how effective a treatment is. In Figure 4, we have compared overall survival rate (%) for different antibodies namely PD-L1 [27], Pembrolizumab [28], Nivolumab [29], and Durvalumab. In the above figure, you can see that the OS (%) rate of antibody Durvalumab is higher than other antibodies.

The overall response rate (ORR %) is a direct measure of medication tumoricidal efficacy and is described as the proportion of patients who show a partial or complete response to treatment. It excludes stable illness. In Figure 5, we have compared the overall response rate (%) for different antibodies namely Placebo [4], Nivolumab (28), and Durvalumab. In the above figure, you can see that the ORR (%) rate of antibody Durvalumab is higher than other antibodies.

## 4. Result and Discussion

In this study, we have discussed and found that durvalumab's Progression-Free Survival (PFS %) rate, overall survival (OS %) rate, and overall response (ORR %) rate are higher than other antibodies. Hence, it is a cost-effective therapy following chemoradiation therapy. In individuals with unresectable stage III enhanced solid tumors, durvalumab as consolidation therapy is presently the benchmark of therapy following chemoradiation therapy. durvalumab consolidation therapy could be cost-effective for individuals with unresectable advanced solid cancers who have not advanced after final chemoradiation therapy, according to our findings. Provided the significant impact that immunotherapy medications are projected to have on tumor costs, it is critical to identify areas where such therapies are most effective. The overall survival rates for different antibodies were also compared which revealed a better survival rate for the durvalumab antibody. Ultimately, we think that durvalumab could be securely utilized for the treatments of numerous solid tumors depending on our meta-analysis of safety and effectiveness and that its usage in conjunction with tremelimumab warrants further investigation. Durvalumab consolidation therapy is the most recommended and cost-efficient therapy. Sometimes it may cause some side effects such as vomiting, stomach cramps, declining interest in food, fatigue, joint spasms, cough, breathlessness, common cold-like blocked nose, sneezing, dry cough, pain when urinating, loss of hair, redness, or bloating in your arms and legs. If any patient has an allergic reaction or any side effects described above, they should get emergency medical help. Further research should be focused on identifying maybe through enhanced biomarker selections the number and proportion of patients who respond positively to immunotherapy and have the minimum side effects, as this will minimize the overall cost burden incurred by these expensive treatments. Several studies testing durvalumab and consolidation therapy for other solid tumors are now underway or enrolling participants, which will give new information in the future.

## Figures and Tables

**Table 1:** Patient data characteristics.

No. of patients	Age	Gender	Stage of the cancer
1	64	Male	IIIC
2	49	Male	IVA
3	38	Female	0
4	32	Male	IIB
5	54	Male	IV
6	49	Female	IIIA
7	60	Female	IIA
8	42	Female	I
9	31	Male	IIA
10	48	Female	IV
11	62	Male	IVA
12	58	Female	IIIB

## Data Availability

The data and materials used to support the findings of this study are included within the published article.
